# Numerical Study of Cell Cryo-Preservation: A Network Model of Intracellular Ice Formation

**DOI:** 10.1371/journal.pone.0058343

**Published:** 2013-03-20

**Authors:** Wei Li, Geer Yang, Aili Zhang, Lisa X. Xu

**Affiliations:** 1 School of Biomedical Engineering, Shanghai Jiao Tong University, Shanghai, China; 2 Med-X Research Institute, Shanghai Jiao Tong University, Shanghai, China; University of California, Berkeley, United States of America

## Abstract

In this study, a new intracellular ice formation network model, coupled with an improved cell dehydration model has been developed. The non-uniform dehydration of the cell during freezing is simulated with moving boundary condition. Internal cell structures like cell nucleus are taken into consideration. The IIF network model is developed from classic diffusion limited IIF model in order to simulate spatial ice growth pattern inside cells. Simulation results suggest that cell nuclear plays a significant role in cryo-dehydration and would affect water/CPA concentration gradient inside the cell. At the same time, the ice growth pattern of exogenous IIF hypothesis is examined in the model. It is consistent with our previous experiments, in which we witnessed the intracellular ice first grown into the nucleus before spreading to the whole intercellular space. According to this model, the water concentration difference between nucleus and cytoplasm during cryo-dehydration could partly explain why ice crystal in the nucleus grows faster. However, it is not the dominate factor. Higher diffusion coefficient in cell nucleus might play a more important role in the phenomenon.

## Introduction

Cryopreservation has been an important method for storage of various cells and tissues [1]–[6], mechanisms of freezing induced cell damage have been studied in details [7], [8]. Previous research shows during fast freezing, water locked in the cell induces lethal intracellular ice formation (IIF) [9]–[11]. On the other hand, the cell dehydrates in slow cooling. The highly condensed cryo-protective agent (CPA) and other solutes would also endanger the cell [12], [13].

In recent years, computational modeling has been introduced to find optimal cooling procedure. Following the founding work of Mazur et al. [7] and Toner et al. [14], Kalsson et al. built a classic diffusion-limited model for Intracellular ice formation (IIF) simulating both homogeneous and heterogeneous nucleation in a biological cell [15]. Zhao et al. improved this model so it can provide reasonable intracellular information for both higher and lower CPA concentrations [16]. This model was further improved by Chen et al. by taking the effect of soft impingement in crystal growth computation [17]. Yang et al. improved this model with free volume models [18] and studied the cell type dependency of intracellular ice formation [19]. Yet, most of the aforementioned works have treated the cell as a homogeneous droplet in a dimensionless control volume. These models neglected the non-uniformity of water distribution inside cell during cryo-dehydration, and cannot reflect the spatial growth pattern of intracellular ice formation. In intracellular water/CPA transportation modeling, earlier researches also treated the cell as a homogeneous droplet. This quasi-steady approach could be justified by the relative small size of cell in comparison with diffusion and convection terms. However, dehydration could still induce water/CPA concentration gradient from cell nucleus to cytoplasm, given reduced diffusivity in lower temperature. Intracellular ice formation can be affected by this gradient. There have been laudable 2-D and 3-D cryo-dehydration models published to examine the water/CPA concentration gradient in cell, but the unique property of the cell nuclear has not been taken into consideration [20], [21], [22]. As the volume fraction of free water inside the nucleus can be twice as much as that in cytoplasm [23], its effect on both intracellular ice formation and transportation may not be neglected.

Moreover, as showed by our previous experiments, the intracellular ice would start from one point on the cell membrane, creep through the cytoplasm into the nuclear, and then grow rapidly throughout the nucleus before it spreads into other cytoplasm region [24]. Therefore, it would be interesting to see whether water/CPA concentration or other different bio-physical property between cytoplasm and nucleus would affect the growth pattern of intracellular ice. In this paper, a novel network model describing intracellular ice growth is developed, coupled with a 3-D mass transportation model. The spatial IIF growth pattern in cell is simulated and the impact of cell nuclear in both mass transportation and intracellular ice growth are studied.

### Mass transportation model

**Figure 1:**
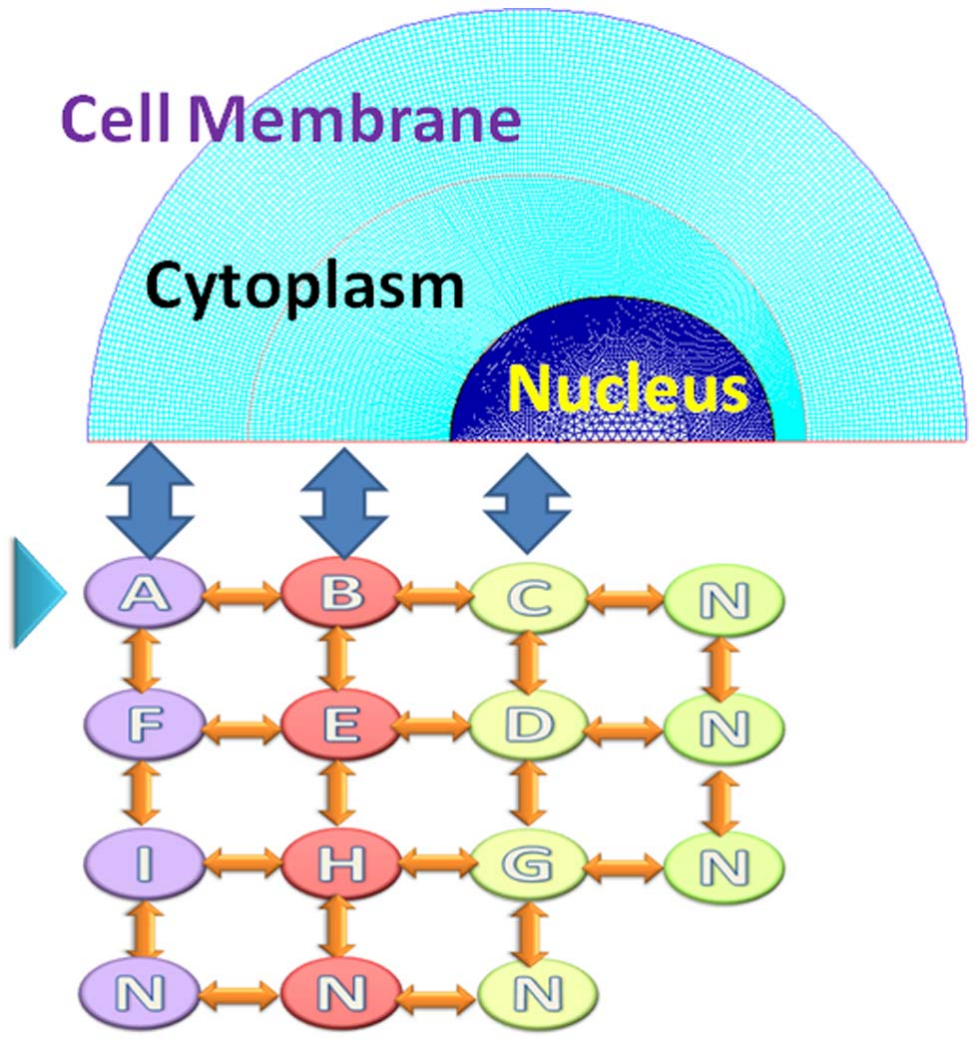
Schematic of the oocyte cell model. The upper part is the mesh graph of the transportation model. The lower part is the IIF network model. Two models are coupled together by sampling and interpolation during iterations. A, F, I are the nodes in cytoplasm adjacent to cell membrane, G, D, C are the nodes in the cell nuclear. H, E, B is nodes in cytoplasm near nuclear membrane.

The oocyte cell is simplified to be an ellipsoid with the dimensional parameters listed in Table 1. The schematic of the model is shown in Figure 1. The cell is divided into two domains (the cytoplasm and the nucleus) with different physical properties. The cytoplasm is enclosed by the semi-permeable plasma membrane. A permeable nuclear envelop separates the nucleus from the cytoplasm.

**Table 1:** Geometric parameters of oocyte model.

Parameter	Symbol	Value(μm)
semi-major axis of cell	l_maj_	41.2
semi-minor axis of cell	l_min_	39
semi-major axis of nuclear	s_maj_	15.15
semi-minor axis of nuclear	s_min_	12.99
Thickness of nuclear envelop and peripheral area	b_env_	0.1
radius of nucleus	r_neu_	6.22
displacement of nucleus from origin	d_nlr_	8

#### 1. Water transportation across the cell membrane

During freezing, the cells are surrounded by the extracellular cryo-protective solution. The local water-loss flux 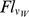 across the cell membrane is estimated as follows [7], [15], [18]:
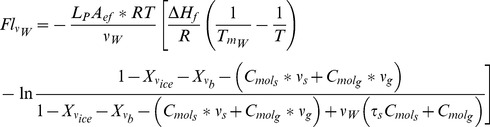
(1)


where *L_p_* is the membrane water permeability, *A_ef_* is effective membrane surface area per unit volume for transport; *R*, gas constant; *T*, temperature; *ΔH_f_*, specific fusion heat of water; *T_mw_*, equilibrium melting point of water; *ν_w_*, *ν_s_* and *ν_g_* are specific volumes of water, salt and glycerol; 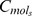 and 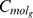 are local mole concentrations of salt and glycerol per unit volume. *X_vb_* represents the inactive volume fraction in the local cytoplasm; 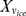 represents the local ice fraction. For details, please refer to Table 2–5.

**Table 2:** Parameters used in free volume model for pure water [18], [19].

Parameter	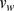 (mol^−1^ml)	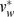 (g^−1^ml)	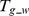 (K)	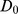 (m^2^s^−1^)	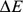 (m^2^s^−1^)	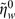 (Pa s)	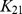 (K)	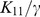 (g^−1^ K^−1^ml)
Value	18	0.91	136	1.39×10^−7^	1.98×10^3^	3.33×10^−5^	−19.73	1.945×10^−3^

**Table 3:** Parameters used in the free volume models for CPA (glycerol) [18], [19]

Parameter	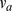 (mol^−1^ml)	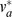 (g^−1^ml)	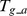 (K)	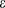	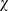	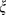
						
Value	73.3	0.716	192.15	0.55	0.29	0.92
Parameter	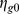 (Pa s)	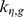 (ml^−1^mol)	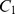	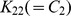 (K)	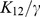 (g^−1^ K^−1^ml)	*W*
Value	1.44×10^11^	7.4×10^−2^	17.4	30.12	5.93×10^−4^	0.427

**Table 4:** Modification parameters of the oocyte model [25]–[33].

Parameter	Symbol	Value(μm)
Diffusion rate in cytoplasm	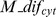	0.3
Diffusion rate in nucleus	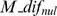	0.78
Diffusion rate in nuclear envelop and peripheral area	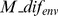	0.078
Porosity in cytoplasm	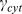	0.45
Porosity in nucleus	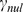	0.85
Equivalent Porosity in nuclear envelop area	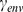	0.2

**Table 5:** Parameters used in the IIF model for mouse oocyte [18], [19].

Parameter	Symbol	Value
Exogenous heterogeneous nucleation area	*A_e_exo_*,	64416.476
	*A_e_sal_*,	64416.476
	C	0
	M	1
	*d*	0.02
Initial salt concentration	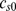 (M)	0.142
Membrane permeability reference value	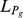 (m^2^ s kg^−1^)	7.26×10^−15^
Membrane permeability activation energy	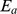 (J mol^−1^)	5.57×10^4^
Membrane permeability reference temperature	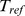 (K)	273.15
Homogeneous nucleation rate kinetic coefficient	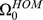 (s^−1^m^−3^)	2.0×10^50^
Homogeneous nucleation rate thermodynamic coefficient	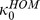 (K^5^)	1.1×10^12^
Heterogeneous nucleation rate kinetic coefficient	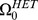 (s^−1^m^−2^)	3.56×10^8^
Heterogeneous nucleation rate thermodynamic coefficient	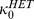 (K^5^)	4.6×10^9^
Specific heat of fusion of water	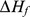 (J mol^−1^)	6016.52
Equilibrium melting point of water	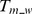 (K)	273.15

Due to cryo-dehydration, the cell membrane shrinks and the simulation boundary moves inward. To define its movement, a speed vector is computed at each section on cell membrane. The local membrane movement is set to be perpendicular to the membrane surface. And the membrane surface is assumed to be always smooth. The face movement speed vector *v_s_* could be determined from the local water flux across the cell membrane: 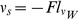
(2)


#### 2. Transportation in the cytoplasm and nuclear

In the cytoplasm, previous researchers have found that the diffusivity and solutes concentration are not uniform inside the cell [23], [25]–[28]. Some experiments suggest that a large portion of water in the cytosol is excluded from solvent process [29], [30]. Meanwhile, there is a significant higher fraction of water mutually soluble with glycerol in the nucleus, which indicates a larger volume fraction of “free water” in the nuclear [23]. Considering the existence of the cytoskeleton and other cellular organelles, the cytoplasm is approximated as porous media [31]. Its liquid phase consists of “free water” and other free diffusive materials. Its “inactive part” is composed by the cytoskeleton, organelles and congregated macro-molecules. The “bounded water” and other discrete part of solutes are also considered to be inactive part. Bounded water does not actively take part in intracellular transportation, due to their low mobility [27], [28].

In order to address the diffusion in the highly concentrated cytoplasm solution (possibly in glassy state at low temperatures), equations from free volume model are used to determine the mutual diffusion rate in bulk solution 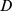 [18].
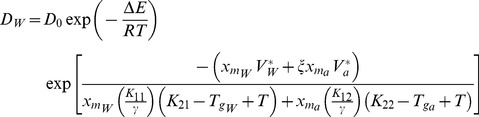
(3)

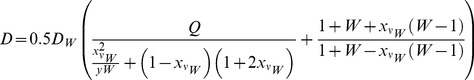
(4)

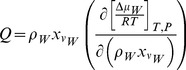
(5)

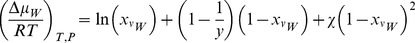
(6)


Where *D_W_* is the self-diffusion coefficient of water in aqueous glycerol solution; *x_vw_* is the water volume fraction in the system; *D_0_* is the pre-exponential factor, *ΔE* is the activation energy for a water molecule to overcome the attractive forces from the surrounding molecules, *ξ* is the ratio of the molar volume of water to that of glycerol, and *K_12_* and *K_22_* are two free volume parameters for glycerol. *χ* is the Flory–Huggins interaction parameter of the glycerol-water solution system, *y* is the ratio of specific volume of glycerol to water, and *W* is the ratio of the self-diffusion coefficient of glycerol to water in an infinitely dilute glycerol-water solution. *T_gw_* and *T_ga_* represent the glass transition temperatures of pure water and glycerol, respectively. For details please see to Table 2 and Table 3.

The effective diffusion rate *D_eff_* in the cytoplasm and cell nuclear is much slower than that in bulk solution, because of the longer traverse distance as well as the “crowding” effect in highly concentrated solutions [25]–[28], [30], [32]. It is determined by the porous medium theories,
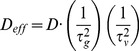
(7)


where *τ_g_* represents the geometry effect on traverse distance of porous media; and *τ_ν_* represents the hindering coefficient of crowding effect. *D* is the diffusion rate in bulk solution. Since it is hard to obtain the exact values of *τ_g_* and *τ_ν_* for the cytoplasm, the effective diffusion rate in the cytoplasm (*D_eff_*) is estimated as: 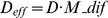
(8)


where 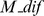 is the gross diffusion modification factor. It has certain values for different cell regions. As mentioned in previous paragraph, cell nucleus has higher free water concentration and exhibits high solubility with glycerin comparing to cytoplasm [23]–[30]. Besides, as the cytoplasm contains a large portion of bounded water, and the cell nucleus has a comparatively higher water/glycerin mobility [33], [34], the water diffusion coefficient in the nucleus is assumed to be higher than that in the cytoplasm. Values of the parameters are listed in Table 4.

The main governing equation of transportation in the porous medium is:
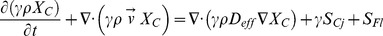
(9)


where *γ* represents the local porosity; *ρ* is the density of media; *X_c_* is the volume fraction of media. *S_Cj_* and *S_Fl_* are source terms from IIF and cross membrane water loss at the cell boundary.

#### 3. Transportation across the nuclear membrane

Transportation of the glycerin and water across the envelope is assumed to follow free diffusion law considering the existence of pores on the nuclear envelope. Previous research on nuclear envelop reported a high permeability in 34°C, a decreased permeability in 10°C, and then a return to high permeability in 2°C [35]. This observation suggests the nuclear envelope may have lost its selective permeability when at the cryo-preservation temperatures. Its permeability is assumed to be depended on its physical structure [36].
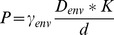
(10)


where *γ_env_* is equivalent porosity of the nuclear envelop, *D_env_* is the effective diffusion rate on nuclear envelop region, *K* is the compatibility between the molecule and the nucleus membrane material, *d* is the thickness of the membrane region.

### Intracellular IIF Network model

In order to understand the ice formation direction inside the cell during cooling, a node network is built to predict the crystal growth in different parts of the cell as shown in Figure 1. Nodes A, F and I are the points in the cytoplasm near the plasma membrane, Nodes C, D and G are positioned in the nucleus, while nodes B, E and H locate in the middle of the cytoplasm. The concentrations of water, salt and CPA, the diffusion rate, the porosity and the viscosity at these nodes are sampled from the intracellular transportation model and they would be fed into the IIF network model for computation. After the transient ice volume change obtained, the water-to-ice volume fraction change would be interpolated and returned back to the transportation model. When ice forms in one part of the cell, the concentration of water in that region is:
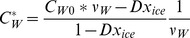
(11)where *ν_w_* is molar volume of water. 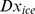 is the local ice volume growth.

To calculate the IIF growth in each node, the classic IIF equations developed by Yang et al. [19] are used. The local ice fraction per unit volume is determined by [19]: 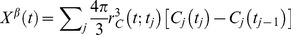
(12)where *r_c_* is the radius of ice nucleus at time *t* of an ice crystal nucleated at time *t_i_*. It is decided by diffusion-limited crystal growth model [19].
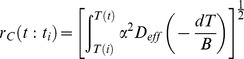
(13)
*α* is a dimensionless parameter for ice growth, and *D_eff_* is effect mutual diffusion coefficient of the intracellular solution to be estimated from free volume models [18], [19].

And for each unit volume in the cytoplasm domain, the number of ice crystals per unit volume *C_j_* could be calculated as [19]

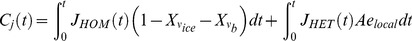
(14)where *Ae_local_* is the local effective area for heterogeneous ice formation per unit volume, and it is decided by the summation of the heterogeneous nucleation area. *X_νice_* and *X_νb_* are the local volume fraction of ice and local volume fraction of inactive part. Homogenous *J_HOM_(t)* and heterogeneous nucleation rate *J_HET_(t)* per unit volume can be obtained from [14]:
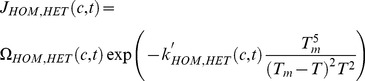
(15)where *c* represents the local concentrations of solution. Ω*_HOM_* and Ω*_HET_* are the kinetic parameter for homogeneous and heterogeneous IIF. *κ'_HOM_* and *κ'_HET_* are the thermodynamic parameter of homogenous and heterogeneous IIF. *T_m_* is the equilibrium melting point of the solution. The details of the parameters are listed in Table 5.

The total crystallized volume fraction can be modified using the following equations [17], [37]: 
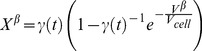
(16)where *γ(t)* is the initial super-saturation which can be calculated as follows [38]

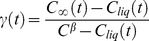
(17)where *C_∞_(t)* is the concentration of water in the bulk cytosol, *C_liq_(t)* is the liquid water concentration at the given temperature.

According to the exogenous triggering IIF hypothesis, when freezing starts, the heterogeneous IIF area *A_e_* at all nodes are set to be zero, and an exogenous heterogeneous nucleation area 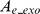 is introduced to node A and triggers its heterogeneous IIF. The growth of ice volume in node A would further cause ice nucleates to spread into the surrounding nodes and providing a heterogeneous nucleation area *A_e_* for nodes B and F. The spreading of heterogeneous nucleation area generated from one node to another is assumed to follow:
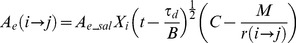
(18)where *X_i_* is the volume fraction of ice at node i, *r(i→j)* is the distance between node i and node j. *A_e_sal_*, *C*, *M* and *τ_d_* are the parameters to be fitted.

For each node exclude A, the intracellular heterogeneous *A_e_* for ice formation resulted from crystal growth of adjacent nodes is then determined by:
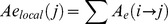
(19)


where *i* are the nodes adjacent to *j* in the network.

For node A, apart from the exogenous heterogeneous nucleation area 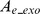 given at the beginning, the growth of the ice volume is also affected by the adjacent nodes, thus the 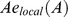 is:
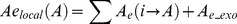
(20)


The definition and values of the parameters are listed in Table 5.

## Results and Discussion

The above proposed transportation model is computed by Fluent together with embedded C program. A mutual diffusion process is computed between glycerol, salt and water. An axisymmetric mesh-map with three major domains is plotted (see Figure 1) to reflect the mass transportation model of cell. The temperature is assumed to be uniform throughout the cell.

Cell shrinkage is controlled by Eq.2 and is enabled by smoothing, stratification and re-meshing. Water loss across the semi-permeable cell membrane described by Equation 1 is simulated by adding water loss source term on the membrane. The IIF computation is iterated by four-order Runge-Kutta method. IIF induced water loss and mass diffusion is computed by four-order-implicit format. Ice fraction in transportation model is treated as inactive volume. To couple the transportation model and IIF network together, 3-order interpolation method is used.

### 1. Water concentration difference as a relationship with temperature

**Figure 2:**
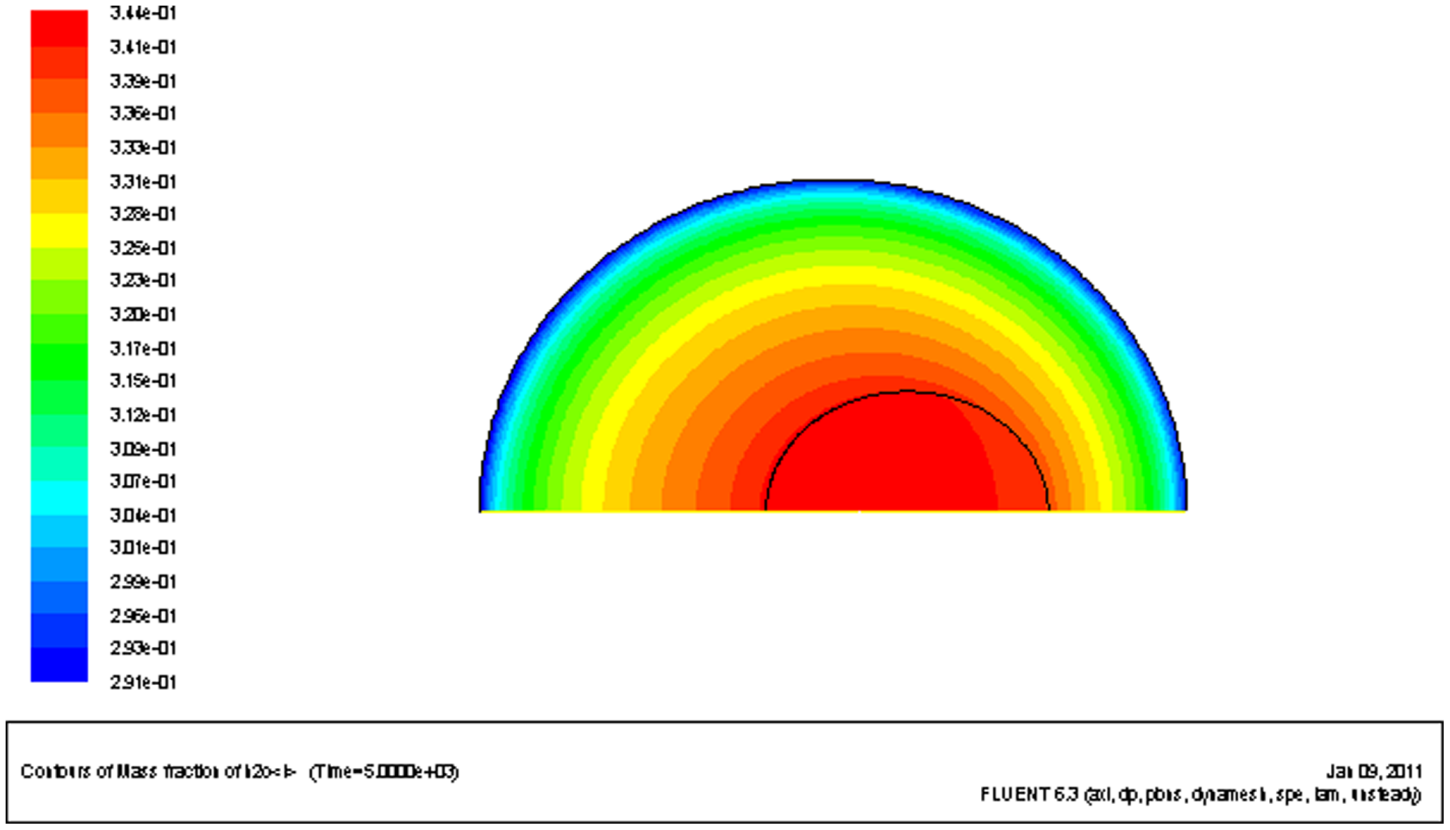
Contour map of water fraction in the cell. Sampled at −67.144°C. Initial CPA concentration at 6 mol/L, Cooling rate at 0.01 K/s.

**Figure 3:**
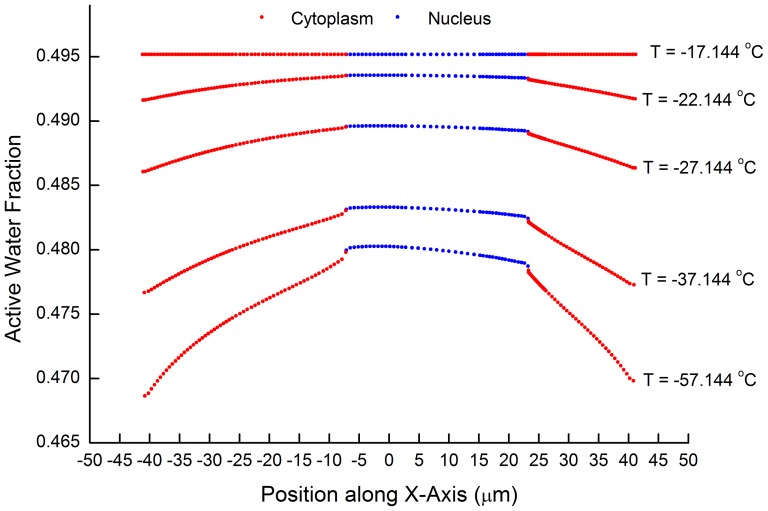
Change of water fraction distribution along x-axis with temperature. Sampled at −17.144, −22.144, −27.144, −37.144, −57.144°C. Initial CPA concentration at 6 mol/L, Cooling rate at 0.1 K/s.

During cryo-dehydration, a water/CPA concentration gradient is formed in the cell. Before significant intracellular ice formation happens, the water concentration in the center of cell is higher than that on the periphery (See Figure 2). As shown in Figure 3, the water/CPA gradient becomes greater as the temperature drops. This is because the diffusion rate of the solutes in the solution decreases greatly when frozen with a high CPA concentration, and the water would be locked in the cell center which increases the water/CPA concentration gradient between cell nucleus and cell membrane. Though at the same, the cross-membrane water loss rate decreases due to the decreased membrane permeability. When the local water dehydration and the diffusive transport reaches equilibrium, the concentration gradient remains constant as shown in Figure 3. Moreover, when the cooling rate is greater, the water/CPA concentration difference between the nucleus and the cytoplasm would become even more significant (results not shown).

### 2. Cell structure's effect on water concentration distribution

**Figure 4:**
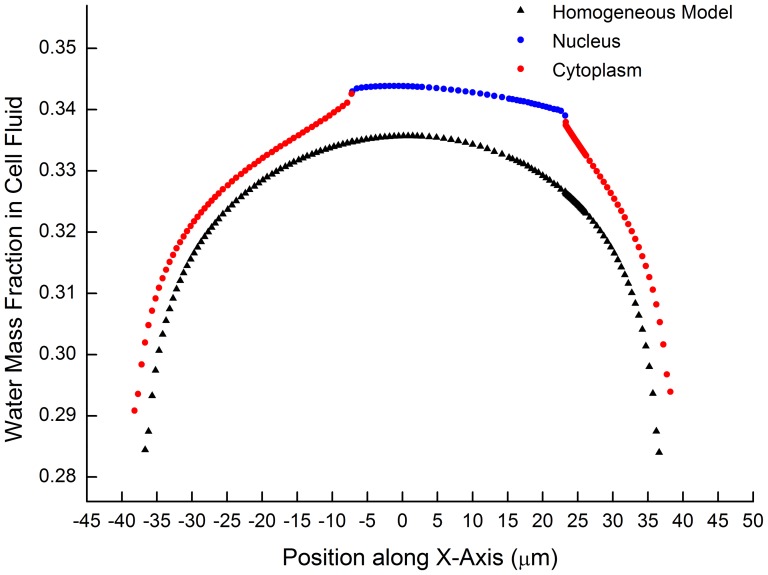
Comparison of water distribution obtained by this model and by ideal solution model. The upper curve is obtained by the proposed model while the lower one is result from the ideal solution model (without nucleus and nuclear envelope considered). Initial CPA  = at6 mol/L, Cooling rate at 0.01 (K/s), Sampled at −67.144°C.

A comparison is made between the results obtained by this model and the ideal solution model (without nucleus and nuclear envelope). The intracellular water concentration distribution calculated from both models is illustrated in Figure 4. It shows that both models have observed a high water concentration in the center of the cell. When the cell nuclear (higher curve) is considered, there is a more significant concentration gradient and the dehydration is slower. Given the similar physical parameters used, the relatively lower effective diffusion rate comparing to the ideal solution model may be the reason of these differences. Meanwhile, the difference of free water volume fraction (in the form of porosity) and diffusivity between nucleus and cytoplasm may also plays an important role. As the probability of the intracellular ice formation is related to the water content of the cell, the exaggeration of the water loss by former models may consequently down-estimate the possibility of IIF.

### 3. Comparison of intracellular ice growth rate between the nucleus and the cytoplasm

**Figure 5:**
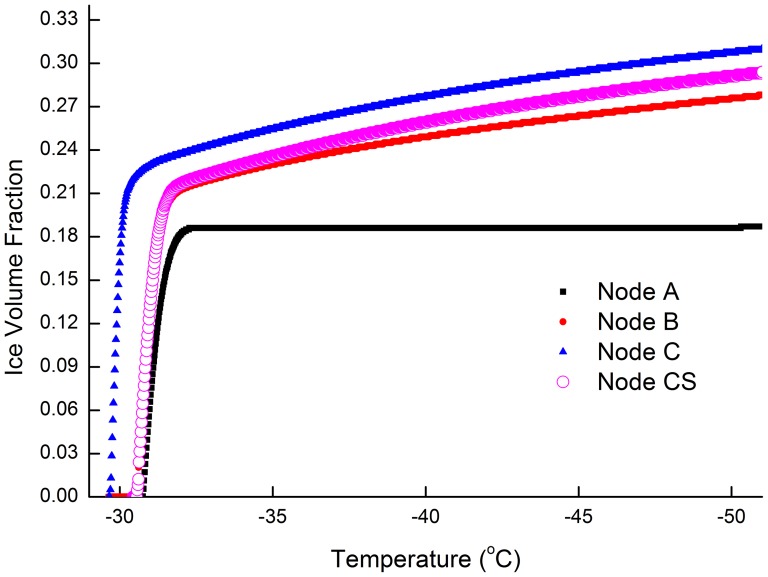
Comparison of ice crystal growth rate between nucleus and cytoplasm. The ice growth rate is compared among four nodes represent different parts of the cell (Nodes C and CS are same node in the nucleus with different diffusion setting, node B is in the middle of cytoplasm, node A is adjacent to the cell membrane). The gross diffusion modification factor for node C is set to the value as in the cell nuclear (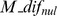), while the gross diffusion modification factor for species in Node CS is set to the value as in the cytoplasm (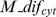). A heterogeneous nucleation area A_e_ is equally given to all nodes inside cell. Initial CPA at 4.8 mol/L, Cooling rate at 0.02 (K/s). Maximum step length at 0.004 K.

As it has been shown in Figure 4, the cell nucleus has a higher water concentration during cryo-dehydration. To find out whether this difference would affect the intracellular ice growth, the intracellular ice volume growth rate has been obtained and results shown in Figure 5. The ice growth pattern is compared among four nodes represent different parts of the cell (Nodes C and CS are same node in the nucleus with different diffusion setting, node B is in the middle of cytoplasm, node A is adjacent to the cell membrane). Each node receives a constant heterogeneous IIF area 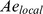 simultaneously when cooling starts. Moreover, to find out how diffusion rates would have affected the ice crystal growth, the gross diffusion modification factor for node C is set as it should in cell nuclear (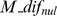), while the gross diffusion modification factor in Node CS is set to the value as in the cytoplasm (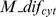) (see Table 4). As shown in the figure, the ice growth in node B is much faster than in node A while the ice growth in node CS is a little faster than that in node B. The limit gradient of water/CPA concentration between node A, B and CS appears to contribute insignificantly in ice volume growth. The results suggest that the difference of water concentration caused by cryo-dehydration alone would not decisively alter the ice crystal growth rate. On the other hand, the Figure shows that ice growth in node C is much faster than that in node CS, this indicates the diffusion rate plays a important role in deciding IIF growth rate.

### 4. Intracellular ice growth in network model

**Figure 6:**
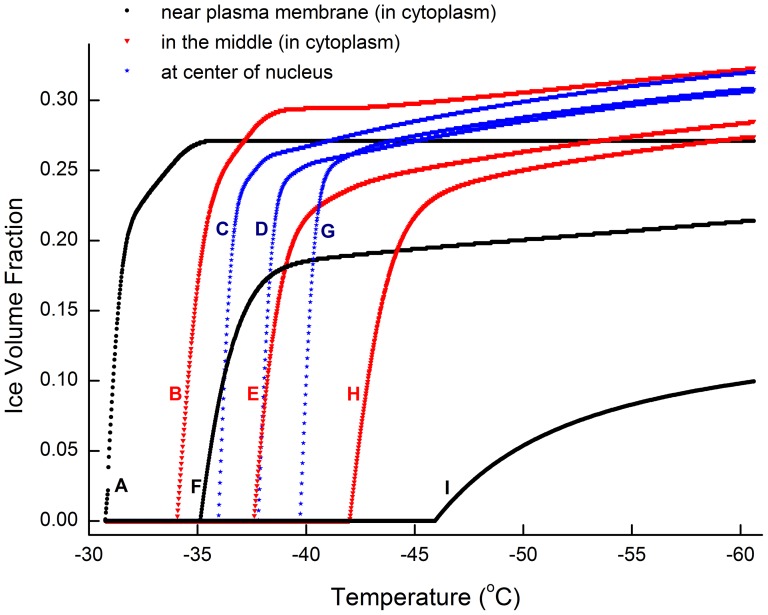
Growth of intracellular ice volume in the network. Initial CPA at 4.8 mol/L, Cooling rate at 0.02 (K/s). Maximum step length at 0.004 K.

**Figure 7:**
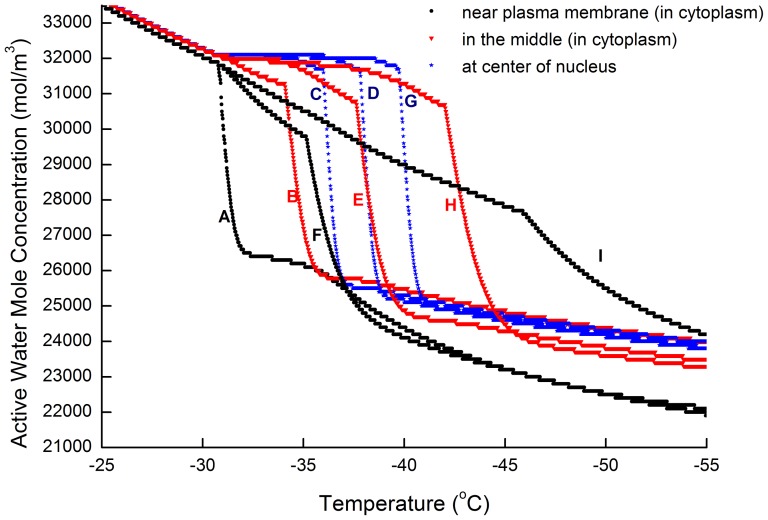
Water molar concentration development in the network. Initial CPA at 4.8 mol/L, Cooling rate at 0.02 (K/s), Maximum step length at 0.004 K.

The intracellular ice volume growth rate and the correspondent water concentration at each node are obtained with the IIF network model and results shown in Figure 6 and 7. Seen from Figure 6, when an exogenous heterogeneous nucleation area 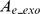 is given at the node A at the beginning, the network model reveals that the ice would more likely to grow into the nucleus (node C, D, G) before overspreading into cytoplasm (node H, I). The IIF sequence at the beginning is A-B-F, since B and F is adjacent to A. At −36°C, the ice growth at node C (the first nucleus node) begins to surpass that of node F, this is because nodes in the nucleus (node C, D, G) initially has a higher water concentration and overall diffusion coefficient. Shortly after the intracellular ice grows into notable volume in node B and node F, heterogeneous IIF in node E begins to grow rapidly. At the same time, ice growth in node C also provides a heterogeneous IIF area for node D and its ice volume begins to rise rapidly. At −38°C the ice volume in node D begins to surpass that in the node E, showing the ice grows much more quickly in the nucleus comparing to that in the cytoplasm. Node E and node G turns plateau almost at the same time with node G's ice volume fraction a little higher. This marks the moment when ice fills the nucleus. These results are in accordance with our former experimental observations [24].

As shown in Figure 7, the water concentration in all nodes decreases with time. If without ice formation, these curves would turn plain at a lower temperature, as dehydration across the cell membrane becoming slower due to low permeability. According to this figure, when water start to turn ice, water concentration in that icing node and those nodes surround it would see a dramatic reduction (water in surrounding nodes would diffuse into the icing node). From the figure, it can be seen that the water concentration difference caused by cryo-dehydration are insignificant comparing to the rapid water loss when icing starts.

### 5. Intracellular ice growth in network model devoid of specific diffusion modification

**Figure 8:**
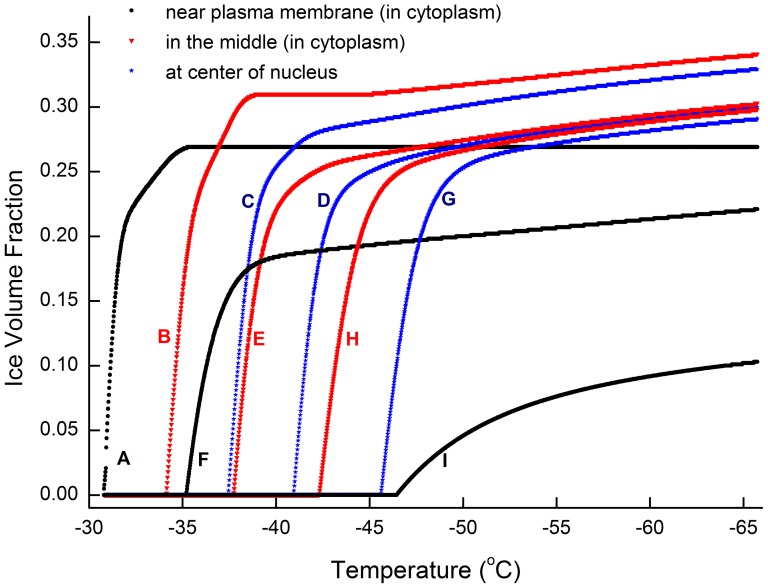
Growth of intracellular ice volume in the network (same gross diffusion modification factor). Diffusion coefficient difference between the nucleus and the cytoplasm is not accounted (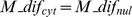). Initial CPA at 4.8 mol/L, Cooling rate at 0.02 (K/s), Maximum step length at 0.004 K.

In order to study the impact of diffusion rate on the spatial IIF pattern, the effect of gross diffusion modification factors (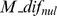 and 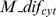) has been examined. As shown in Figure 8, when gross diffusion modification factor in nucleus and cytoplasm is made equal (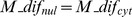), the network model reveals that ice forms in the nucleus is not earlier than that in the cytoplasm. As in the above case, significant icing happens accordingly at nodes A, B and F. However, the ice growth in the nucleus nodes is not decisively faster. In the end, all three nodes in the middle of cytoplasm (B, E, and H) turn plateau earlier than their counter partner in the nucleus(C, D, G). The node I, which is adjacent to the other side of the cell membrane, is still the last to icing.

**Figure 9:**
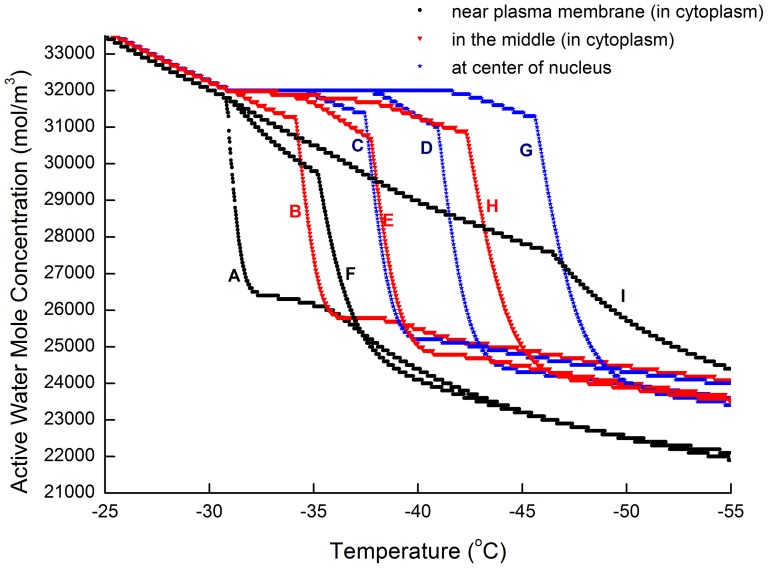
Water molar concentration development in the network (same gross diffusion modification factor). Diffusion coefficient difference between the nucleus and the cytoplasm is not accounted (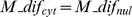). Initial CPA at 4.8 mol/L, Cooling rate at 0.02 (K/s), Maximum step length at 0.004 K.

Figure 9 also deviates significantly from Figure 7 due to different icing sequence. Although water concentration and ice growth is mutually dependent, we find that the dehydration induced water/CPA gradient is insignificant comparing to that generated by ice formation. It also suggests that the dehydration induced water/CPA concentration difference may not be a dominate factor in determining the intracellular ice growth direction. The diffusion rate difference between the cell nucleus and the cytoplasm may be more important.

## Conclusions

In this research, an intracellular mass transportation model has been built to study the species transportation and distribution inside cells. A diffusion limited network model of intracellular ice formation is established to simulate the spatial intracellular ice growth direction during freezing. The coupled mass transportation and IIF network model has enabled us to analyze ice growth pattern inside cell during cooling and attested the experimental findings which has found the intracellular ice first grows into the nucleus before spreading to whole cell. The unique structure and constitution of nucleus made it a much more preferable IIF sites than the cytoplasm. Higher diffusion rate may be one of the contributing factors as showed in this study. As some of the parameters at low temperatures lack of direct experimental support, further study is needed to improve the model.
